# The psychometric properties of the subscales of the GHQ-28 in a multi-ethnic maternal sample: results from the Born in Bradford cohort

**DOI:** 10.1186/1471-244X-13-55

**Published:** 2013-02-15

**Authors:** Stephanie L Prady, Jeremy NV Miles, Kate E Pickett, Lesley Fairley, Karen Bloor, Simon Gilbody, Kathleen Kiernan, Rachel Mann, John Wright

**Affiliations:** 1 Department of Health Sciences, University of York, York, UK; 2 RAND Corporation, Santa Monica, USA; 3 Bradford Institute for Health Research, Bradford Teaching Hospitals NHS Foundation Trust, Bradford, UK; 4 Department of Social Policy and Social Work, University of York, York, UK

**Keywords:** Born in Bradford, Psychometric evaluation, Antenatal anxiety and depression, Postnatal anxiety and depression, Multi-ethnic, Ethnic minority

## Abstract

**Background:**

Poor maternal mental health can impact on children’s development and wellbeing; however, there is concern about the comparability of screening instruments administered to women of diverse ethnic origin.

**Methods:**

We used confirmatory factor analysis (CFA) and exploratory factor analysis (EFA) to examine the subscale structure of the GHQ-28 in an ethnically diverse community cohort of pregnant women in the UK (N = 5,089). We defined five groups according to ethnicity and language of administration, and also conducted a CFA between four groups of 1,095 women who completed the GHQ-28 both during and after pregnancy.

**Results:**

After item reduction, 17 of the 28 items were considered to relate to the same four underlying concepts in each group; however, there was variation in the response to individual items by women of different ethnic origin and this rendered between group comparisons problematic. The EFA revealed that these measurement difficulties might be related to variation in the underlying concepts being measured by the factors.

**Conclusions:**

We found little evidence to recommend the use of the GHQ-28 subscales in routine clinical or epidemiological assessment of maternal women in populations of diverse ethnicity.

## Background

Good maternal mental health is important for a child’s future health and wellbeing as depression and other mental health problems can interfere with bonding, attachment, enrichment activities and parenting behaviour [1,2]. Children of mothers who suffer from depression are more likely to experience behavioural problems and have lower school attainment; this can set a child on a pathway of fewer life chances with associated risks of health problems [3-7]. Antenatal distress, particularly anxiety, and postnatal depression are strongly correlated [8,9]; however, screening presents challenges as normal physical and hormonal changes may interfere with the sensitivity and specificity of screening instruments, particularly those containing items relating to somatic symptoms which will naturally be disturbed by both pregnancy and caring for an infant [10,11].

Commonly used population screens for psychological distress include the General Health Questionnaire (GHQ) family of instruments. The 28-item version (GHQ-28) was developed in the 1970’s from a factor analysis of the GHQ-60 to distinguish four correlated underlying concepts as factors, each comprised of seven items related to the presence of somatic symptoms (subscale A, items 1–7), anxiety and insomnia (B, 8–14), social dysfunction (C, 15–21) and severe depression (D, 22–28) [12].

The GHQ-28 has been translated into several languages and used internationally. A key concern when applying a screening instrument in a different population is that it might perform unexpectedly; therefore ‘emic’ measures that have intrinsic meaning in the culture and populations in which they will be used [13,14] are preferable in the development of mental health measures. ‘Etic’ development of mental health measures whereby translated and/or transplanted measures are applied to a population under the assumption that concepts are similar across cultures may not be of particular concern when the health of a single population is assessed; however, potential variation has consequences when assessing differences between populations. If differences exist in the way groups interpret the underlying concept being measured, or variation in the strength of relationship between a question about a symptom and the concept, and this goes unnoticed or ignored, it might be difficult to distinguish between true variations (or similarities) in mental health, and spurious findings. Johnson [15] highlights the complexities inherent when defining and operationalising cross-cultural equivalence, with interpretive differences of concepts and constructs nested in lexical, semantic and idiomatic variation. Factors that can affect instrument accuracy include population variation in mental illness prevalence [16], differences in the strength of association between the items and the implied factor being measured, variation in the expression of psychological symptoms, and systematic differences in how the response scales for each question are completed [17].

Several methods are available to explore potential differences and test hypotheses to examine if measures are equivalent across populations. For multi-dimensional instruments the number of factors being measured by the items can be derived from exploratory factor analysis (EFA). The same technique can be employed to determine which items are most strongly (or weakly) related to the factors/s and which items relate to multiple factors. The instrument’s equivalence across different populations can be tested using confirmatory factor analysis (CFA) which can indicate whether a factor is associated with the same item set across groups (configural invariance), the strength of the relationship between each item and the factor is the same across groups (metric invariance), and whether both groups have a similar response to an item response scale (scalar invariance). Such analyses lead to the development of a measurement model in which equivalence of the scale’s performance in each group is suggested or rejected either from the observed data or after correction for systematic differences.

Using EFA, the four-factor structure of the GHQ-28 has been found to vary between countries, and across populations there may be less distinction between subscales A (Somatic) and B (Anxiety and Insomnia) than originally found [18]. Fewer studies have explored the performance of the GHQ-28 subscales during or after pregnancy; however, an analysis of a Yoruban translation given to pregnant Nigerian women indicated that subscales A and B and the more cognitive (non-suicidal ideation) items from subscale D represented a single factor [19]. Large scale investigations into the scale’s performance in maternal populations and in ethnic minority women are lacking.

The GHQ-28 was used as a measure of maternal psychological distress for the Born in Bradford community birth cohort ( http://www.borninbradford.nhs.uk) which includes roughly equal size populations of White women and those of South Asian descent. Because of the potential for variation in the underlying concepts measured by the GHQ-28 between ethnic groups and languages of administration, and due to the maternal characteristics of the cohort, we examined its psychometric properties to ensure that cohort-wide comparisons were valid between all subpopulations.

We aimed at identifying a strategy that could be used to measure and compare symptom subscale scores during and after pregnancy for women of varying cultural backgrounds and for those completing the GHQ-28 in different languages.

## Methods

### Population

Born in Bradford (BiB) is a longitudinal multi-ethnic birth cohort study aiming at examining the impact of environmental, psychological and genetic factors on maternal and child health and wellbeing [20]. Bradford is a city in the North of England with high levels of socio-economic deprivation and ethnic diversity. Women were recruited prior to a glucose tolerance test offered as a routine procedure to all pregnant women registered at Bradford Royal Infirmary at 26–28 weeks gestation. A baseline questionnaire was administered to women who consented via an interview conducted in a designated room with semi-private booths. Women could choose to have their interview conducted in either English, Mirpuri (a spoken variant of Punjabi) or Urdu. Women not able to converse in any of these three languages were eligible to enrol but did not complete the baseline questionnaire and thus are not included here. The full BiB cohort recruited 12,453 women during 13,776 pregnancies between 2007 and 2010 and the cohort is broadly characteristic of the city’s maternal population. Ethical approval for the data collection was granted by Bradford Research Ethics Committee (Ref 07/H1302/112).

Two samples from the BiB cohort were used to explore the properties of the GHQ-28. First we report on data from 5,299 women with singleton births enrolled between November 2007 and March 2009 who completed the phase two version of the three versions of the baseline questionnaire. Second, we used a subset of the cohort, known as BiB1000, to assess the structure of the GHQ-28 in pregnancy and postnatally. BiB1000 participants in our sample were enrolled between August 2008 and March 2009, completed the phase two baseline questionnaire and consented to repeat visits at six, 12, 18, 24 and 36 months postpartum. We report on the antenatal and six-month GHQ-28 data for 1,305 women with singleton births.

### GHQ-28

An initial Urdu translation of the GHQ-28 questionnaire was adapted for use as a script in this population by a professional translator through a process of refinement using participatory methods [21,22]. Assessment of understanding was undertaken with groups of bilingual then monolingual Urdu women from local Children’s Centres. A Mirpuri version was transliterated from a second draft that used a similar iterative process with bilingual then monolingual Mirpuri speaking women. Scripts were finalised from the third draft version in each language.

The GHQ-28 was administered on paper as part of a self-completion module at the end of the interview for women who chose to complete their baseline questionnaire in English. For the women who chose Mirpuri or Urdu language, the GHQ-28 questions were read aloud and the research assistant coded the response on paper. Verbal administration was necessary because there is no written form of Mirpuri, and not all Urdu speakers are fluent in reading and writing the Urdu language. Some of the women were accompanied; therefore verbal responses may have been audible to the accompanying person. For the women in BiB1000, the six-month GHQ-28 was administered in the women’s home by research staff in the language of choice.

The GHQ-28 has a 4-item response scale anchored (typically) with ‘Not at all’, ‘No more than usual’, ‘Rather more than usual’, and ‘Much more than usual’. Several scoring options are available; we used the Likert method to indicate symptom severity, which scores the item response between 0–3 (0–1–2–3, subscale range 0 to 21) as this is the recommended method for assessment of the subscales. We excluded the few cases where either the GHQ-28 was missing in its entirety, or did not contain at least one intact subscale.

### Ethnicity

Questions relating to ethnicity in BiB were based on those used in the UK’s 2001 census and comprised of one question that asked which ethnic group the mothers considered they belonged to (White, Mixed ethnic group, Black or Black British, Asian or Asian British, Chinese or other), followed by a further question, based on their response, about their cultural background. For example, if a participant selected ‘Asian or Asian British’ as ethnic group, a choice of cultural background could be selected from the following; Indian, Pakistani, Bangladeshi, Indian Caribbean, African-Indian. Self-defined ethnic and cultural group information was taken from the baseline questionnaire and classified into the two most numerous groups of White and Pakistani; all other responses were coded into a separate category (Other). The few cases of women identified as mixed White and Pakistani (N = 18 in the cohort) were classified in the White group. Due to the low number of non-UK born White women (N = 146) we did not further differentiate the cultural background of those who identified as White.

### Language of administration

The interviewer recorded the language in which the interview was conducted.

### Analysis

We tested for measurement equivalence on the subscales by multi-group confirmatory factor analysis (CFA), using Mplus version 7 with a robust maximum likelihood (MLR) estimator as our data were not normally distributed. MLR is a full information estimator that employs all the available data and thereby calculates unbiased parameter estimates in the presence of data which are missing at random or missing completely at random [23]. Some women completed the instrument on more than one occasion due to multiple pregnancies. This introduces non-independence into the sample, which can lead to incorrect values for standard errors and fit statistics (fit statistics based on chi-square). We accounted for this minor clustering of the full cohort data by utilising a sandwich estimator (the cluster command within Mplus, combined with the complex samples approach). We fitted increasingly restrictive pairwise models in five subpopulations; women who completed the questionnaire in English for the ethno-cultural groups of Pakistani, White and Other, women who completed the questionnaire in Mirpuri (Pakistani and Other), and women who completed it in Urdu (Pakistani and Other). As a subscale score is calculated independently from other subscales in practice, we considered the fit of each subscale separately for each subpopulation, with no cross-loading items permitted. If a factor was not associated with the same item sets across groups (i.e. configural invariance was not met) a model generation strategy was used where items within subscales were removed until adequate fit was achieved for each subpopulation for the same items for each factor. We considered model fit adequate if thresholds for three indices were met; comparative fit index, CFI (≥0.95), root mean square error of approximation, RMSEA (≤0.08) and standardised root mean square residual, SRMR (≤0.06). We interpreted modification indices to help identify the most problematic items and accepted the solution that retained the largest number of items, for the best fit, across groups. If configural invariance was then indicated, we tested whether the strength of the relationship between each item and the factor were equal across groups by constraining factor loadings to be equal across both groups (metric invariance). If metric invariance was indicated we then tested for scalar invariance by also constraining item intercepts to be equal [24-26]. For analysis purposes the latent variable is assigned the scale of the first item. If there is variation in how each group responds to an item response scale, a unit change in a factor score will be associated with an unequal change in the score of an item across groups. The presence of this Differential Item Functioning (DIF) indicates that between group comparisons will be invalid [27].

We treated the data as continuous for analysis purposes. Likert data can be treated as continuous, or can be considered to be ordered categorical (i.e. an item response theory – IRT-based approach). There is debate in the literature regarding the most appropriate method for analysing such data [28,29] however our aim was to analyse the scales in the same metric in which they are employed. The scales are typically scored by summing (or equivalently averaging) items, not scored using IRT-based methods, hence we analysed the covariance matrix.

We repeated this process (configural, metric, scalar testing) on the subsample of women who completed the measure both during pregnancy and six-months postpartum (BiB1000). We restricted the BiB1000 analysis to those women who completed both questionnaires in the same language. Two women from the ‘Other’ ethnic groups did not complete the questionnaire in English and only three women completed the GHQ-28 in Mirpuri. Therefore, our analysis compared these data across four ethnic groups; English administration for White women, English (Pakistani), English (Other) and Urdu (Pakistani).

As noted previously, we considered model fit adequate if thresholds for three indices were met; CFI (≥0.95), RMSEA (≤0.08), and SRMR (≤0.06). We did not interpret change in *χ*^2^ as an indicator of invariance in increasingly restrictive models as it is relatively insensitive to change in large samples. Instead we used a change in CFI of ≤0.01 together with a change in SRMR of ≤0.03 to indicate substantive invariance, setting the SRMR criterion to ≤0.01 when evaluating scalar invariance [30,31].

As the same seven items were not associated with the same factors across groups, i.e. configural invariance was not indicated, we followed up the CFA of the BiB cohort with exploratory factor analysis (EFA). We specified an EFA with between 1 and 8 latent variable solutions as implemented in Mplus. To determine the most parsimonious solution that best fit the data we examined the scree plot [32] for the point of inflexion and used the fit criteria detailed above.

## Results

### Description of sample

#### BiB cohort

We excluded 176 (3.3%) women without at least one GHQ-28 subscale score, along with a further 34 (<1%) women where the language of administration was not documented. Of the remaining 5,089 cases, 2.3% were missing a minor amount of GHQ-28 data. Nearly all the women who completed the questionnaires in a language other than English were born outside of the UK, and around 10% of the Mirpuri and 7% of the Urdu questionnaires were completed by women of Other ethnic origin (Table 1).

**Table 1:** Population characteristics, BiB Cohort

**Language of administration and ethnic group**	**English (White)**	**English (Pakistani)**	**English (Other)**	**Mirpuri (Pakistani & Other)**	**Urdu (Pakistani & Other)**	**Total**
	**N = 2104 (41.3%)**	**N = 1480 (29.1%)**	**N = 626 (12.3%)**	**N = 219 (4.3%)**	**N = 660 (13.0%)**	N = 5089* (100%)
Age at recruitment (years), mean (SD)	26.5 (6.1)	27.3 (4.9)	28.3 (5.5)	28.1 (5.6)	27.6 (5.2)	27.2 (5.6)
Cohort baby is first child, N (%)	1,023 (48.6)	511 (34.6)	265 (42.3)	54 (24.7)	188 (24.5)	2,041 (40.1)
Born in UK, N (%)	1,962 (93.3)	1,014 (68.5)	270 (56.5)	4 (1.9)	8 (1.3)	3,258 (66.5)
Age at migration for non-UK born (years), median (IQR)	22 (15 to 25)	17 (4 to 21)	24 (19 to 27)	21 (19 to 24)	21 (19 to 24)	21 (18 to 24)
*Antenatal GHQ-28 scores*						
Total score, mean (SD), median (IQR)**						
Likert method	22.9 (9.9)	26.2 (11.7)	24.7 (11.8)	19.3 (8.5)	21.5 (9.4)	23.7 (10.7)
	22 (16 to 29)	25 (17 to 34)	23 (16 to 31)	18 (13 to 24)	20 (14 to 27)	22 (16 to 30)
GHQ method	5.4 (4.9)	7.0 (5.9)	6.3 (5.9)	4.5 (4.3)	5.9 (4.6)	6.0 (5.3)
	4 (1 to 8)	6 (2 to 11)	5 (2 to 9)	3 (1 to 7)	5 (2 to 9)	5 (2 to 9)
≥6 (GHQ method), N (%)	788 (39.4)	681 (50.7)	259 (45.5)	60 (29.3)	290 (45.6)	2,078 (43.7)
*missing total score, N (%)*	106 (5.0)	136 (9.2)	57 (9.1)	14 (6.4)	24 (3.6)	306 (6.6)
Subscale scores (Likert), median (IQR)						
A Somatic symptoms	6 (4 to 9)	8 (5 to 11)	7 (4 to 10)	7 (4 to 9)	8 (5 to 11)	7 (4 to 10)
B Anxiety and Insomnia	7 (3 to 10)	8 (4 to 11)	7 (3 to 10)	4 (1 to 7)	4 (1 to 8)	6 (3 to 10)
C Social dysfunction	7 (7 to 9)	8 (7 to 10)	8 (7 to 10)	7 (7 to 8)	7 (7 to 9)	8 (7 to 9)
D Severe depression	0 (0 to 2)	1 (0 to 3)	1 (0 to 3)	1 (0 to 1)	1 (0 to 1)	0 (0 to 2)

* Includes those with at least one intact GHQ-28 subscale and the language of administration, N presented may not total 5089 due to small amounts of missing data, ** total scores have more missing data but are not used in the analysis, *SD* standard deviation, *IQR* interquartile range.

#### BiB1000

Of the 1,305 women enrolled, 186 (14.3%) were not included as they did not use either Urdu or English at each administration, and a further 24 were missing GHQ-28 data. The characteristics of women recruited to the BiB1000 study did not appear to differ markedly from the main cohort (Table 2).

**Table 2:** Population characteristics, BiB1000

**Language of administration and ethnic group**	**English (White)**	**English (Pakistani)**	**Urdu (Pakistani)**	**English (Other)**	**Total**
	**N = 469 (42.8%)**	**N = 369 (33.7%)**	**N = 103 (9.4%)**	**N = 154 (14.1%)**	N = 1095* (100%)
Age at recruitment (years), mean (SD)	27.0 (6.1)	27.2 (4.8)	28.2 (5.9)	28.8 (5.5)	27.4 (5.6)
Cohort baby is first child, N (%)	229 (48.8)	132 (35.9)	27 (26.5)	64 (41.6)	452 (41.4)
Born in UK, N (%)	463 (98.7)	248 (67.4)	0	66 (55.5)	777 (73.4)
Age at migration for non-UK born (years), median (IQR)	3 (1 to 3)	17 (4 to 21)	21 (19 to 25)	23 (16 to 26)	20 (12 to 24)
*Antenatal GHQ-28 scores*					
Total score, mean (SD), median (IQR)**					
Likert method	23.4 (10.1)	26.3 (11.7)	24.1 (9.2)	25.1 (12.1)	24.7 (10.9)
	22 (16 to 29)	25 (18 to 34)	23 (18 to 28)	24 (15 to 32)	23 (17 to 31)
GHQ method	5.6 (0.23)	6.9 (0.32)	7.1 (0.45)	6.5 (0.48)	6.3 (0.17)
	5 (2 to 8)	6 (2 to 11)	6 (4 to 10)	5 (1 to 11)	5 (2 to 9)
≥6 (GHQ method), N (%)	194 (43.1)	180 (52.0)	59 (58.4)	68 (46.0)	501 (47.9)
*missing total score, N (%)*	19 (4.1)	23 (6.2)	2 (1.9)	6 (4.0)	50 (4.6)
Subscale scores (Likert), median IQR					
A Somatic symptoms	7 (4 to 9)	8 (5 to 10)	10 (7 to 13)	7 (4 to 10)	7 (5 to 10)
B Anxiety and Insomnia	7 (4 to 10)	7 (4 to 11)	5 (2 to 9)	7 (3 to 11)	7 (4 to 10)
C Social dysfunction	7 (7 to 9)	8 (7 to 10)	8 (7 to 9)	8 (7 to 10)	8 (7 to 9)
D Severe depression	0 (0 to 2)	1 (0 to 3)	0 (0 to 1)	1 (0 to 3)	0 (0 to 2)
*Postnatal GHQ-28 scores*					
Total score, mean (SD), median (IQR)**					
Likert method	15.9 (9.2)	17.2 (10.2)	16.6 (9.3)	15.4 (9.0)	16.3 (9.5)
	13 (9 to 20)	14 (10 to 22)	14 (10 to 21)	13 (9 to 20)	14 (10 to 21)
GHQ method	2.4 (3.9)	3.0 (4.3)	3.9 (4.4)	2.3 (3.6)	2.7 (4.1)
	1 (0 to 3)	1 (0 to 4)	2 (1 to 6)	1 (0 to 3)	1 (0 to 4)
≥6 (GHQ method), N (%)	72 (16.0)	63 (18.6)	26 (26.5)	23 (15.8)	184 (17.8)
*missing total score, N (%)*	20 (4.3)	30 (8.1)	5 (4.9)	8 (5.2)	63 (5.8)
Subscale scores (Likert), median IQR					
A Somatic symptoms	4 (2 to 6)	5 (3 to 7)	6 (4 to 8)	3 (2 to 6)	4 (2 to 7)
B Anxiety and Insomnia	3 (1 to 6)	3 (1 to 7)	3 (0 to 6)	3 (0 to 6)	3 (1 to 6)
C Social dysfunction	7 (6 to 7)	7 (5 to 7)	7 (5 to 7)	7 (5 to 7)	7 (6 to 7)
D Severe depression	0 (0 to 1)	0 (0 to 1)	0 (0 to 1)	0 (0 to 1)	0 (0 to 1)

* Includes those with at least one intact GHQ-28 subscale from each time point and the same language of administration both times, N presented may not total 1095 due to small amounts of missing data, ** total scores have more missing data but are not used in the analysis, *SD* standard deviation, *IQR* interquartile range.

### Confirmatory factor analysis, BiB cohort

#### Model generation strategy

Generally there was little evidence of good fit of the items to each subscale across groups. To achieve adequate fit across the sample all subscales required item reduction (Table 3). The best fit was not always achieved for the same cluster across subpopulations, this was marked for subscales C (Social Dysfunction) and D (Severe Depression). The retained GHQ-28 questions are provided in Table 4.

**Table 3:** Fit of complete scales and model generation results

***Groups***	***Subscale***	***Somatic (A)***		***Anxiety & insomnia (B)***		***Social dysfunction (C)***		***Severe depression (D)***	
	***Fit indices***	***Items 1–7***	***Items 1–4***	***Items 8–14***	***Items 10–13***	***Items 15–21***	***Items 15–19***	***Items 22–28***	**Items 23–26**
*English (White)*	*χ*^2^(df)	1039 (14)	13 (2)	311 (14)	4 (2)	105 (14)	20 (5)	632 (14)	32 (2)
	CFI	**0.688**	0.993	**0.938**	0.999	**0.946**	0.986	**0.719**	**0.963**
	RMSEA	**0.187**	0.051	**0.100**	0.020	0.054	0.037	**0.145**	**0.085**
	SRMR	**0.096**	0.014	0.040	0.006	0.032	0.020	**0.084**	0.026
*English (Pakistani)*	*χ*^2^(df)	660 (14)	5 (2)	315 (14)	3 (2)	79 (14)	37 (5)	186 (14)	6 (2)
	CFI	**0.733**	0.996	**0.914**	0.999	0.965	0.972	**0.898**	0.995
	RMSEA	**0.177**	0.035	**0.120**	0.016	0.056	0.067	**0.091**	0.034
	SRMR	**0.080**	0.011	0.051	0.007	0.028	0.023	**0.052**	0.014
*English (Other)*	*χ*^2^(df)	232 (14)	2 (2)	140 (14)	0.5 (2)	38 (14)	10 (5)	102 (14)	19 (2)
	CFI	**0.782**	1.0	**0.908**	1.0	0.965	0.986	**0.879**	0.945
	RMSEA	**0.158**	0.0	**0.120**	0.0	0.053	0.041	**0.100**	**0.117**
	SRMR	**0.075**	0.009	0.051	0.004	0.032	0.025	0.052	0.034
*Mirpuri (Pakistani and Other)*	*χ*^2^(df)	75 (14)	1 (2)	30 (14)	6 (2)	20 (14)	4 (5)		1.2 (2)
	CFI	**0.802**	1.0	**0.927**	0.956	0.962	1.0	*	1.0
	RMSEA	**0.141**	0.0	0.073	**0.091**	0.045	0.0		0.0
	SRMR	**0.067**	0.13	0.051	0.031	0.045	0.025		0.002
*Urdu (Pakistani and Other)*	*χ*^2^(df)	153 (14)	3 (2)	126 (14)	9 (2)	83 (14)	9 (5)	26 (14)	9 (2)
	CFI	**0.838**	0.997	**0.884**	0.983	**0.844**	0.982	0.960	**0.937**
	RMSEA	**0.123**	0.027	**0.110**	0.072	**0.086**	0.033	0.036	0.072
	SRMR	**0.062**	0.012	0.055	0.023	0.055	0.028	0.043	0.037
*Comments*			Mirpuri and Urdu best fit for items 1–5		Several other models were a better fit for Mirpuri and Urdu		The full item set (15–21) fit all groups best except Urdu		24–27 fit all groups best except Mirpuri which was poor (CFI = 0.701, RMSEA = 0.163)

Adequate fit statistics were considered to be CFI ≥0.95, RMSEA ≤0.08 and SRMR ≤0.06, bolded fit indices indicate less than satisfactory fit, * severe model estimation difficulties.

**Table 4:** GHQ-28

**Have you:**	**Item retained for CFA**
**Subscale (A) Somatic**	
**1. Been feeling perfectly well and in good health?**	**Yes**
**2. Been feeling in need of a good tonic?**	**Yes**
**3. Been feeling run down and out of sorts?**	**Yes**
**4. Felt that you are ill?**	**Yes**
5. Been getting any pains in your head?	No
6. Been getting a feeling of tightness or pressure in your head?	No
7. Been having hot or cold spells?	No
**Subscale (B) Anxiety and Insomnia**	
8. Lost much sleep over worry?	No
9. Had difficulty in staying asleep once you are off?	No
**10. Felt constantly under strain?**	**Yes**
**11. Been getting edgy and bad-tempered?**	**Yes**
**12. Been getting scared or panicky for no good reason?**	**Yes**
**13. Found everything getting on top of you?**	**Yes**
14. Been feeling nervous or strung-up all the time?	No
**Subscale(C) Social dysfunction**	
**15. Been managing to keep yourself busy and occupied?**	**Yes**
**16. Been taking longer over the things you do?**	**Yes**
**17. Felt on the whole you were doing things well?**	**Yes**
**18. Been satisfied with the way you’ve carried out your tasks?**	**Yes**
**19. Felt you are playing a useful part in things?**	**Yes**
20. Felt capable of making decisions about things?	No
21. Been able to enjoy your normal day-to-day activities?	No
**Subscale (D) Severe depression**	
22. Been thinking of yourself as a worthless person?	No
**23. Felt that life is entirely hopeless?**	**Yes**
**24. Felt that life isn’t worth living?**	**Yes**
**25. Though of the possibility that you might make away with yourself?**	**Yes**
**26. Found at times you couldn’t do anything because your nerves were too bad?**	**Yes**
27. Found yourself wishing you were dead and away from it all?	No
28. Found that the idea of taking your own life kept coming into your mind?	No

#### Invariance testing

There appeared to be metric invariance between all subpopulations for all reduced item subscales (Table 5). There was evidence of differential item functioning across many of the group comparisons on all subscales, which indicated that some subpopulations used the item response scales differently under the same state of mental health as measured by the latent factor. For example, in the comparison between the English (Pakistani) and Mirpuri groups which failed the invariance test of the reduced Somatic subscale, a one unit change of the latent variable (on a 4-point scale) resulted in a change in item 3 of 0.39 of a point greater on a 4-point scale in the English group than the Mirpuri group. For the comparison between the invariant English (Pakistani) group and the English (Other) group, this difference was just 0.07 for the Pakistani group.

**Table 5:** Invariance testing on reduced GHQ-28 item subscales for the BiB Cohort

	***English (Pakistani)***	***English (White)***	***English (Other)***	***Mirpuri (Pakistani & Other)***
*Reduced Somatic subscale A (Items 1–4)*				
*English (White)*	L: 0.001, -0.006			
	**I: 0.024, -0.016**			
*English (Other)*	L: 0.000, -0.005	L:- 0.001, -0.001		
	I: 0.008, -0.021	I**: 0.020, -0.015**		
*Mirpuri (Pakistani & Other)*	L: 0.002, -0.008	L: 0.000, -0.003	L: 0.000, -0.008	
	I**: 0.033, -0.029**	**I: 0.024, -0.022**	I**: 0.065, -0.044**	
*Urdu (Pakistani & Other)*	L: 0.009, -0.019	L: 0.008, -0.017	L: 0.006, -0.017	L: 0.003, -0.014
	I**: 0.033, -0.012**	I: 0.016, -0.009	I**: 0.054, -0.021**	I**: 0.062, -0.035**
*Reduced Anxiety and Insomnia subscale B (Items 10–13)*				
*English (White)*	L: 0.001, -0.003			
	L: 0.007, 0.017			
*English (Other)*	L: 0.000, -0.001	L: 0.001, -0.004		
	L: 0.000, 0.005	I**:** 0.012, -0.007		
*Mirpuri (Pakistani & Other)*	L: 0.002, -0.002	L: 0.002, -0.004	L: 0.001, -0.003	
	I**: -0.027, 0.043**	I: -0.004, 0.018	I**: -0.014, 0.028**	
*Urdu (Pakistani & Other)*	L: 0.020, -0.008	L: 0.019, -0.013	L**:** 0.031, -0.005	L: -0.005, -0.006
	**I: -0.029, 0.053**	**I: 0.013, 0.017**	I: **0.025, 0.032**	I: 0.005, -0.004
*Reduced Social Dysfunction subscale C (Items 15–19)*				
*English (White)*	L: 0.006, -0.015			
	I: 0.004, 0.002			
*English (Other)*	L: -0.002, -0.003	L: 0.002, -0.008		
	I: 0.004, -0.002	I: 0.011, -0.006		
*Mirpuri (Pakistani & Other)*	L: -0.003, -0.006	L: 0.003, -0.010	L: -0.002, -0.012	
	I: 0.015, -0.009	I**: 0.019, -0.010**	I: 0.021, -0.009	
*Urdu (Pakistani & Other)*	L: 0.000, -0.011	L: 0.007, -0.016	L: 0.003, -0.008	L: -0.009, -0.004
	I: 0.019, -0.004	I: 0.030, -0.007	I: 0.021, -0.007	I: **0.016, -0.011**
*Reduced Severe Depression subscale D (Items 23–26)*				
*English (White)*	L: 0.002, -0.010			
	I**: 0.062, -0.052**			
*English (Other)*	L: -0.003, -0.002	L: -0.008, -0.003		
	I: 0.008, -0.006	I**: 0.054, -0.032**		
*Mirpuri (Pakistani & Other)*	L: 0.001, -0.008	L: 0.001, -0.004	L: -0.008, -0.005	
	**I: 0.022, -0.017**	I: 0.006, 0.000	I**: 0.067, -0.023**	
*Urdu (Pakistani & Other)*	L: 0.003, -0.008	L: -0.008, -0.004	L: -0.012, -0.007	L: 0.003, -0.008
	I: 0.010, -0.002	I: 0.015, -0.004	I**: 0.070, -0.019**	I: 0.001, -0.002

Numbers indicate change in CFI, SRMR from less restrictive model, bolded items indicate invariance not achieved, L factor loadings constrained to be equal, I intercepts constrained to be equal.

### Exploratory factor analysis, BiB cohort

The results from the CFA suggested greater variability between English and non-English groups than for pairwise comparisons between the White British, Pakistani and women of other ethnicities who completed the questionnaire in English. We hypothesised that this was due to differences in the underlying factor structure between linguistic-cultural groups and used EFA to investigate this possibility. A better fit was indicated for a five factor model over a four-factor for the sample overall and all English groups, and six factors over five for the Urdu and Mirpuri groups. However, the individual items making up these factors appeared to differ (Table 6). Across the cohort there appeared to be two concepts being measured with the somatic questions; one cluster of items relating to generalised somatic symptoms (items 1–4), and one relating to the two items concerning physical symptoms in or on the head (items 5 & 6, dubbed Head Somatics in Table 4). The depression concept was split into two factors for the women who responded to the Mirpuri version of the GHQ-28. Several items did not load onto any factor (factor loading <0.3) or loaded only weakly (<0.4); in particular Items 7 (hot/cold spells) 15 (busy and occupied) and 21 (enjoy normal activities), indicating little relevance to the observed factors in most of the subpopulations.

**Table 6:** Factor structure of the GHQ-28 for the BiB cohort

***Model***	***Fit statistics***	***Factor 1***	***Factor 2***	***Factor 3***	***Factor 4***	***Factor 5***	***Factor 6***
*(1) Whole sample 38.8% variance explained*	*χ*^2^ = 2601 (248)	Anxiety (3.78)	Depression (2.95)	Social Dysfunction (1.82)	General Somatics (items 1–4) (1.41)	Head Somatics (items 5 & 6) (1.67)	--
	**CFI = 0.940**						
	RMSEA = 0.043						
	SRMR = 0.022						
*(2) English (White) 39.9% variance explained*	*χ*^2^ = 1615 (248)	Anxiety (3.41)	Depression (2.91)	Social Dysfunction (2.01)	General Somatics (items 1–4) (1.49)	Head Somatics (items 5 & 6) (1.36)	--
	**CFI = 0.919**						
	RMSEA = 0.051						
	SRMR = 0.026						
*(3) English (Pakistani) 41.1% variance explained*	*χ*^2^ = 952 (248)	Depression (3.32)	Anxiety (3.21)	Social Dysfunction (2.36)	Head Somatics (items 5 & 6) (1.36)	General Somatics (items 1–4) (1.28)	--
	**CFI = 0.948**						
	RMSEA = 0.044						
	SRMR = 0.024						
*(4) English (Other) 38.3% variance explained*	*χ*^2^ = 571 (248)	Depression (3.20)	Anxiety (2.70)	Social Dysfunction (2.13)	Head Somatics (items 5–7) (1.35)	General Somatics (items 1–4) (1.26)	
	**CFI = 0.941**						
	RMSEA = 0.046						
	SRMR = 0.027						
*(5) Mirpuri (Pakistani & Other) 33.2% variance explained*	*χ*^2^ = 352 (225)	Anxiety (2.18)	Social Dysfunction (2.15)	Depression 1 (items 27 & 28) (1.46)	General Somatics (items 1–4, 7) (1.21)	Depression 2 (items 24–26) (1.21)	Head Somatics (items 5 & 6) (1.08)
	**CFI = 0.907**						
	RMSEA = 0.051						
	SRMR = 0.038						
*(6) Urdu (Pakistani & Other) 32.6% variance explained*	*χ*^2^ = 440 (225)	Depression (2.66)	Social Dysfunction (1.79)	General Somatics (items 1–4, 7) (1.41)	Anxiety 1 (items 11–14) (1.30)	Anxiety 2 (items 8–10) (1.03)	Head Somatics (items 5 & 6) (0.94)
	**CFI = 0.943**						
	RMSEA = 0.038						
	SRMR = 0.027						

Factors presented are most parsimonious with adequate fit statistics that do not include trivial factors, numbers in parentheses indicate post-rotation Eigenvalues which were used to calculate the explained variance, bolded fit indices indicate less than satisfactory fit.

The amount of variance in the overall model explained by the factors was low; from 41.1% for the Pakistani (English) group, to 32.6% of the Urdu responses. The Severe Depression and Anxiety and Insomnia factors accounted for the largest proportion of the variance for most of the groups. The exception was for the Urdu sample, where the Anxiety and Insomnia questions did not appear to be a unified concept and accounted for less of the variance.

### Confirmatory factor analysis, BiB 1000

#### Model generation strategy

Fit of the seven items to each subscale (data not shown) and reduced item factors for the smaller sample (BiB1000) was broadly similar to the BiB cohort (Table 7), except for some severe model estimation problems on the reduced Severe Depression subscale (items 23–26).

**Table 7:** Model generation results, BiB1000

	***Subscale***	***Somatic (A)***		***Anxiety and insomnia (B)***		***Social dysfunction (C)***		***Severe depression (D)***	
	***Items***	***1–4***		***10–13***		***15–19***		***23–26***	
***Groups***	***Fit indices***	***Antenatal***	***Postnatal***	***Antenatal***	***Postnatal***	***Antenatal***	***Postnatal***	***Antenatal***	***Postnatal***
*English (White)*	*χ*^2^(df)	3 (2)	7 (2)	0.3 (2)	2 (2)	0.1 (5)	9 (5)	11 (2)	11 (2)
	CFI	0.997	0.985	1.000	1.000	1.000	0.982	0.974	**0.947**
	RMSEA	0.037	0.076	0.000	0.000	0.000	0.039	**0.099**	**0.100**
	SRMR	0.013	0.020	0.004	0.010	0.010	0.030	0.029	0.040
*English (Pakistani)*	*χ*^2^(df)	9 (2)	0.2 (2)	1 (2)	2 (2)	12 (5)	12 (5)	6 (2)	11 (2)
	CFI	0.974	1.000	1.000	0.999	0.980	0.957	0.977	**0.942**
	RMSEA	**0.096**	0.000	0.000	0.016	0.060	0.061	0.070	**0.113**
	SRMR	0.028	0.004	0.009	0.011	0.025	0.038	0.026	0.035
*English (Other)*	*χ*^2^(df)	1 (2)	2 (2)	16 (2)	4 (2)	7.0 (5)	11 (5)	3 (2)	*
	CFI	1.000	1.000	0.959	0.980	0.984	0.935	0.974	
	RMSEA	0.000	0.000	**0.116**	**0.084**	0.050	0.050	0.069	
	SRMR	0.010	0.016	0.035	0.029	0.036	0.046	0.028	
*Urdu (Pakistani)*	*χ*^2^(df)	1 (2)	1 (2)	5 (2)	5 (2)	6 (5)	4 (5)	*	0.1 (2)
	CFI	1.000	1.000	**0.936**	**0.946**	0.956	1.000		1.000
	RMSEA	0.000	0.000	**0.125**	**0.112**	0.040	0.000		0.000
	SRMR	0.015	0.023	0.041	0.039	0.056	0.005		0.011

Adequate fit statistics were considered to be CFI ≥0.95, RMSEA ≤0.08 and SRMR ≤0.06, bolded fit indices indicate less than satisfactory fit; * severe model estimation difficulties.

#### Invariance testing

Although metric invariance held for the antenatal and postnatal analyses, there was evidence of DIF between many of the subpopulations at one or both time points (Table 8). To check that we had not forced items 23–26 into an ill-fitting factor, as this was the best fit for the cohort’s Mirpuri sample which was absent in BiB1000, we repeated the analysis for the better fitting cluster 24–27; however, models then became inestimable for the Urdu sample.

**Table 8:** Invariance testing on reduced GHQ-28 item subscales for BiB1000

	***(Pakistani)***		***English (White)***		***English (Other)***	
	***Antenatal***	***Postnatal***	***Antenatal***	***Postnatal***	***Antenatal***	***Postnatal***
*Reduced Somatic subscale A (Items 1–4)*						
*English (White)*	L: 0.004, -0.010	L: -0.003, -0.010				
	**I: 0.013, -0.012**	I: **0.056, -0.020**				
*English (Other)*	L: 0.004, -0.014	L: 0.000, -0.011	L: 0.003, -0.019	L: -0.001, -0.013		
	I: -0.006, 0.001	I: 0.007, -0.010	I: 0.007, -0.002	I: 0.015, -0.001		
*Urdu (Pakistani)*	L: -0.006, -0.003	L: 0.000, -0.008	L: 0.000, -0.012	L: -0.003, -0.010	L: 0.000, -0.008	L: 0.000, -0.011
	I: **0.017, -0.011**	I: 0.000, -0.004	I: 0.009, -0.007	I: 0.005, -0.004	I: 0.003, -0.017	I: 0.000, 0.000
*Reduced Anxiety and Insomnia subscale B (Items 10–13)*						
*English (White)*	L: 0.000, -0.015	L: 0.002, -0.017				
	**I: 0.034, -0.021**	I: 0.006, -0.004				
*English (Other)*	L: 0.000, -0.003	L: **0.013, -0.031**	L: 0.000, -0.006	L: 0.001, -0.016		
	I: 0.002, -0.009	--	I: 0.000, -0.011	I: 0.010, -0.006		
*Urdu (Pakistani)*	L: 0.001, -0.015	L: 0.003, -0.015	L: 0.006, -0.022	L: 0.005, -0.017	L: 0.006, -0.024	**L: 0.040, -0.038**
	I: **0.020, -0.015**	I: 0.018, -0.008	I: **0.028, -0.016**	I: 0.009, -0.005	I: **0.079, -0.026**	--
*Reduced Social Dysfunction subscale C (Items 15–19)*						
*English (White)*	L: -0.004, 0.013	L: -0.002, -0.011				
	I: 0.003, -0.001	I: 0.004, -0.001				
*English (Other)*	L: 0.010, -0.024	L: -0.002, 0.007	L: 0.001, -0.025	L: 0.012, -0.018		
	I: -0.001, 0.001	I: -0.002, -0.002	I: 0.009, 0.003	I: 0.000, 0.001		
*Urdu (Pakistani)*	L: 0.008, -0.018	L: -0.015, -0.009	L: 0.001, -0.022	L: -0.010, -0.008	L: -0.001, -0.017	L: 0.004, -0.025
	I: **0.028, -0.015**	I: **0.024, -0.003**	I: **0.046, -0.018**	I: 0.020, -0.004	I: **0.055, -0.014**	I: -0.003, 0.000
*Reduced Severe Depression subscale D (Items 23–26)*						
*English (White)*	L: -0.011, -0.003	L: 0.001, -0.007				
	I: **0.033, -0.022**	I: 0.018, -0.003				
*English (Other)*	L: -0.016, -0.008	**L: 0.082, -0.065**	L: -0.006, -0.011	*		
	I: 0.009, 0.004	--	I: **0.022, -0.014**			
*Urdu (Pakistani)*	*	*	*	**L: 0.072, -0.054**--	*	*

Numbers indicate change in CFI, SRMR from less restrictive model, bolded items indicate invariance not achieved, L factor loadings constrained to be equal, I intercepts constrained to be equal, * severe model estimation difficulties.

## Discussion

We conducted an extensive psychometric evaluation of the GHQ-28 subscales in a large community multi-ethnic maternal cohort in the UK. Our results are important because this is the first large scale investigation in both a maternal population and in South Asian women, where there is uncertainty about measurement equivalence of mental health [33-36]. For each subscale an item reduction strategy was necessary to fit all our defined subpopulations, and there was evidence of differential item functioning in many of the pairwise comparisons. Exploration of the factor structure indicates that this was caused by variation in the concepts being measured, with the most obvious differences visible between groups of women who completed the questionnaire in English and non-English. For example, Anxiety and Insomnia in the Urdu respondents and Severe Depression in the Punjabi respondents did not appear to be related to the same item clusters as women of any ethnicity completing the questionnaire in English. The implication is that the meaning of the underlying concepts for some items differs according to language of administration and between ethnic groups; this may be related to any number of factors such as acculturation, translation or cultural differences in concept or interpretation. Our goal was to define a measurement model to compare symptom severity in each domain across subgroups; our findings indicate that due to lack of invariance we cannot recommend such comparisons across this cohort.

Research indicates the concept (if not the nomenclature) of postnatal distress has recognition and relevance globally e.g. [37,38]. However, internal construction of causality, symptom experience and illness resolution can vary greatly between cultures [39]. For example, in one UK study, women originating from the Punjab who had ‘life troubles’ reported symptoms of sadness and grief that tallied with the notion of depression, but conceptualised their problems as an illness manifesting physically as ‘heavy in the heart’ [40]. Notably, there have been few studies exploring the meaning of depression in pregnant, not postnatal, South Asian women.

Given such potential for variation, it is perhaps unsurprising that we found differences in the attribution of a specific symptom to particular construct of mental distress between the groups in our sample. Our results indicated several interesting points between the relationship of symptoms and mental health during the maternal period, and also between ethnic groups.

### Somatic subscale

Irrespective of cultural background, it is common for people with depression to initially present with somatic symptoms e.g. [14,41]. Somatisation of psychological distress is of interest in maternal populations where new and perhaps unfamiliar bodily changes coincide with any onset of distress. Such simultaneous physical and hormonal changes may complicate self and clinical recognition of potential affective distress. For example, somatic dysfunction might be construed as causative of distress, distress could be overshadowed by physical symptoms that may be considered to have more serious implications for the baby’s health, or body symptoms may simply co-exist alongside with distress. Neither is the concept of somatisation uni-dimensional. Simon et al. [41] define three different presentations; patients with psychological distress who initially present somatic symptoms, those distressed who present with medically unexplained somatic symptoms and those who present somatic symptoms and deny psychological distress. Bhui et al. [14] adds a fourth; presentation of somatic symptoms made significantly made worse by feeling low, stressed or anxious. The topic has generated much theoretical interest for South Asian cultures where somatisation has sometimes [42], but not universally [13,41], been reported to be more frequently endorsed as a symptom of depression. Indeed some data indicate that initial presentation with somatic symptoms might be a function of the patient-doctor interaction rather than a cultural phenomenon [41].

Our data show that broadly, across the maternal population, two concepts related to somatic symptomology were evident; the first comprised of generalised somatic symptoms and the second of symptoms related to the head. A principle components evaluation of a non-maternal European sample with rheumatoid arthritis [43] found a similar split in structure, but a study of pregnant Nigerian women [19] reported that all seven somatic items clustered together. Although there are differences in methodology, this indicates that the split between general and specific somatic symptoms may be related to factors other than maternity, or female gender, and in our study these elements appear stable regardless of ethnic background, language of administration or pregnancy/postnatal status. We suggest that this hypothesis is tested in other population samples.

### Anxiety and insomnia subscale

Antenatal anxiety commonly co-occurs with depression and is antecedent to postnatal anxiety and depression [9,44-46], and our EFA implicated this factor as the largest symptom cluster for most groups. However, the invariance testing indicated some significant problems with comparisons involving the Urdu group, which the EFA revealed was likely due to a split in the underlying concept.

### Social dysfunction subscale

For all groups except the Urdu language groups, the concept of Social Dysfunction was related to all its hypothesised items, confirming the findings in a Nigerian antenatal sample [19]. Excluding comparisons with the Urdu group, this factor also appeared to indicate pairwise invariance. However, the clinical relevance of this subscale is not well researched [47], which limits its relevance in distinguishing psychiatric morbidity from the range of normal changes during pregnancy.

### Severe depression subscale

As noted, anxiety and depression are commonly co-morbid and these two GHQ-28 factors are unsurprisingly correlated, although the depression subscale has been found to garner some additional information [47]. Here it is noteworthy that this subscale measures severe depression with three questions relating to suicidal ideation; notably absent are enquiries into dysphoric mood. Measurement of such a dimension is of interest inter-culturally; Bhugra and colleagues have enumerated that in London, young South Asian women are at higher risk for presenting with attempted suicide than White women [48,49] with cultural and family conflict the actual and perceived causes of such attempts [48,50]. However, the utility of this subscale to measure the concept of suicidality might be limited, as although for the antenatal English language and Urdu respondents the questions seemed unified and the factor important, this was not the case in the Mirpuri group, and there was evidence of invariance between groups. Furthermore, only one of the suicidality questions (item 25) was invariant between groups. Model estimation difficulties that may have been related to low endorsement of these severe items precluded analysis of postnatal data.

### Measurement invariance

After reducing items to create factors which appeared to have reasonable fit across all the subpopulations, the iterative process of invariance testing revealed systematic differences in how the different subpopulations rated themselves on the measurement scales. We would be able to solve the problem of systematic differences in scale response if, as in most CFA analyses, there were just two populations to compare; but due to both cultural and language variation we identified five distinct groups, and as the DIF varied within sub-group pairs, systematic correction is unfeasible. While some of the differences are small and would have a negligible impact on mean scores, some differentials are up to half a point (on a four-point scale) which has the potential to lead to spurious conclusions after comparison.

### Postnatal scores

Interpretation of the analysis into any systematic differences in structure between antenatal and postnatal administration were limited due to difficulties with model estimation, particularly in the Severe Depression subscale.

### Strengths and limitations

Our sample is representative of the maternal community in Bradford, and included a large number of South Asian minority women for whom relatively little is known about mental health in pregnancy. Further, we applied a rigorous approach to our analysis; however, our study does have some shortcomings.

#### Ethnic and cultural classifications

We used limited classifications of ethnicity which may be overly general [14,51] and can only serve as a proxy for more defined distinction of culture and custom [52]. Such is the compromise when epidemiological rather than anthropological methods are used to classify people [53]. Analysing at the level of an arbitrary subgroup may lead to category fallacy [42] with loss of subtle individual effects such as acculturation and financial and social resources; indeed there may be as much variation within groups as there is between. In particular, we combined the group of women of all Other ethnicities into one heterogeneous reference group, which limits decomposition by ethnicity and culture. We split our sample into five (BiB cohort) and four (BiB1000) reference groups by ethno-cultural classification and language of questionnaire, although women within these groups were likely to have different levels of acculturation. Without a specific measure of acculturation it is impossible to assess values, beliefs, expectations, norms and practices of the new culture and the extent of their acquisition, and how much retention of original culture is still present [54]. Acculturation may have affected how women answered the GHQ-28 questions, for example it may have imposed some unmeasured variation in our estimates, or it could have potentially explained some of the differences we found.

#### Ethno-cultural instrument adaptation

The participatory translation process was rigorous and the translated versions had good semantic, content and conceptual equivalence to the English instrument. An Urdu translation of the GHQ-28 assessed in a bilingual (English and Urdu) population in Pakistan found reasonable semantic, conceptual and scale validity [55]. However, in our study there was no formal assessment of criterion or technical equivalence, necessary to establish whether the GHQ-28 performs similarly across cultures regardless of administration verbally or via paper, or whether the interpretation of measurement of mental health remains the same when compared to norms of both cultures [56]. We did not know which women were bilingually fluent, if we did we could have used their selection of language as a basis to disentangle any variance associated with the translation from that of cultural differences in interpretation and differential item functioning [57]. Of note, there may have been unmeasured administration bias as the administration to non-English speakers was verbal and responses that were potentially audible to family members or friends accompanying the women may have affected the way these women answered the questions.

#### Methodological limitations

As discussed in the analysis section, we treated Likert scale data as continuous for the purposes of analysis. Whilst this has the advantages that we described in that section it is problematic in that DIF cannot be described in terms of the scoring of the scale [28,29]. However, such an approach may be more appropriate for determining invariance in the underlying psychological constructs. In CFA, one item in a factor must be held constant (mean of 0 and variance of 1), and because this item’s variability is not calculated, it can lead to spurious conclusions of invariance if the reference item is the source of DIF [27]. This may be relevant as we held the first item in any one cluster as the reference item. In addition, the lack of standardised diagnostic interview to confirm or exclude depression is a limitation to the interpretation of assessment of relevance of the subscales to clinical criteria in this maternal population.

## Conclusions

We have conducted a robust analysis of the GHQ-28 subscales in a large, ethnically diverse pregnant population and found problems with measurement equivalence between ethno-language groups. In particular, the concepts of Severe Depression and Anxiety and Insomnia appear to vary between language of administration and ethnic heritage. Our findings are tempered by uncertainty about how much variation is caused by artefact of translation and administration bias, and how much due to cultural differences in interpretation. We recommend that the GHQ-28 subscale scores are not used to conduct between-group comparisons in this cohort, nor in other ethnically diverse pregnant populations either clinically or epidemiologically, although as indicated for some subscales and for some groups they could be used to explore within-group characteristics.

## Competing interests

The authors declare that they have no competing interests.

## Authors’ contributions

SLP, LF, KEP, KK & KB conceived the idea and designed the protocol, which was advised on by SG, RCM and JNVM and JW. SLP undertook the statistical analysis which was overseen by JNVM. All authors contributed to and have approved the final manuscript.

## Pre-publication history

The pre-publication history for this paper can be accessed here:

http://www.biomedcentral.com/1471-244X/13/55/prepub
